# Mechanisms to build research capacity in the rural health workplace: a realist synthesis

**DOI:** 10.3389/fmed.2025.1584904

**Published:** 2025-06-19

**Authors:** David Schmidt, Emma Webster, David Lyle

**Affiliations:** Faculty of Medicine and Health, School of Rural Health (Orange/Dubbo), University of Sydney, Dubbo, NSW, Australia

**Keywords:** distributed training, research education, rural health, workplace-based education, realist synthesis

## Abstract

**Introduction:**

Workplace-based research training contributes to research capability and capacity in rural areas where access to university expertise is limited. Rural health complexities and the diverse approaches previously used to build research capacity have led to a lack of clarity about how to build research capacity within rural health services.

**Methods:**

Using a critical realist foundation, we explored distributed workplace-based rural research training and synthesized five studies centered in rural New South Wales, Australia. Critical realism allowed the exploration of the structural supports and barriers for workplace-based research training activities and the ability of individuals to pursue research activities within rural health workplaces.

**Results:**

The component studies showed that distributed rural research training programs improve individual research capability by developing research skill, increasing research experience and facilitating research networks across sectors. Rural research activities are characterized by individual agency and partnering or relationships to access support and expertise. Structural barriers including a lack of operational planning for research and few ongoing research opportunities limit translation of capability into research capacity.

**Discussion:**

Individual workplace-based research training is effective, but not sufficient to build and maintain research capacity. Structural supports such as organizational commitment and careful training design can maximize cooperative partnerships with education partners. Addressing both structural and individual factors is needed to build rural health research capacity and generate real-world health research to drive meaningful improvements in rural health.

## 1 Introduction

People living in rural and remote locations experience significant health disadvantage when compared to their urban counterparts (1, 2). These disadvantages are linked to disparities in healthcare access (3, 4), fewer specialist doctors and allied health professionals, and specialist services clustered in cities (5). Other health service access challenges include large geographical distance between centers, low population densities (1) and disparities caused by social determinants of health (6, 7).

These challenges highlight the unique circumstances associated with rural and remote healthcare delivery. In Australian healthcare delivery, urban models of care are often applied to rural or remote areas, a “one size fits all” approach that may not translate into rural and remote areas (8, 9). The development of tailored rural solutions and bespoke models of care are required to better meet health needs of these unique populations with a goal of equal opportunity for good health “regardless of location” (10).

Addressing these particular circumstances and needs of rural and remote healthcare delivery in Australia indicates a need for rural-specific research (11, 12) including developing and implementing rural models of care (13) and translating relevant urban research into rural environments (14). Better rural healthcare relies upon a better understanding of the rural healthcare environment and rural health delivery.

One difficulty in understanding rural health issues is the limited number of rurally-based researchers (11, 15). Historically most researchers have been urban-based and if they conducted research in rural areas, this has not led to increased research activity driven by those rural communities (12). Research conducted with, within and by rural health services and rural clinicians has advantages in identifying the critical issues relevant to the rural or remote context and understanding of rural people’s mindset and characteristics (11, 16, 17). The unique circumstances surrounding rural healthcare delivery have led for a call for specific rural training for researchers in Australia (12).

Research training and capacity building within health services has taken many forms over the past two decades, including grant programs (18, 19), partnerships (20, 21), embedded researcher models (22–26) and training programs (27–31). The success of these initiatives is measured using different metrics, including self-rated research experience (28, 32, 33), completion rates (34), research activity (18, 35) and the ability to secure grant funding (21, 36). Other reported metrics include publications or presentations (37, 38), workforce development (29, 39), influence on policy and practice (18, 20) or research confidence (40). Provision of a formal qualification or articulation with research higher degrees was an important feature of some programs (26), but many programs led to no formal qualification. In Australia a range of health disciplines have been targeted for research capacity building, including Aboriginal health workers (39, 41, 42), allied health staff (22, 23, 43–47), medical staff (35, 48) and primary health care workers (20, 33, 49, 50).

The range of learner groups, approaches, contexts and metrics add complexity to understanding the relative merits of each approach. This highlights the need for studies such as this synthesis, where critical realism is used to create clarity from this complexity.

The setting for this synthesis is the public health system New South Wales (NSW), Australia. The NSW public health system is structured with a centralized governance and policy body overseeing 15 Local Health Districts (LHDs) and multiple specialty networks responsible for delivering clinical services (51). Nine of these LHDs cover rural and remote areas (51).

Within these rural LHDs, there has been an effort to build research capacity through the Rural Research Capacity Building Program (RRCBP), a distributed, workplace-based research training program (27–29, 34). This program was created in recognition of limited research expertise in rural health services and within rural health workers (29). The stated aims of the program are to build research knowledge and skill while contributing to the rural research evidence base (34). This aligns closely to the clinician-researcher model, producing rural clinicians with research capability and experience, whilst building research capacity.

The synthesis explores the constraints inherent in the rural health system and what rural research capacity building can achieve within this context. Five papers examining research capacity building within NSW and within the public health system (33, 52–55) were included in this synthesis. Collectively, the studies and a critical realism perspective allow development and testing of generative mechanisms that explain “why things are as they are” in research capacity building in rural health services, with the rural NSW experience as an exemplar. These underlying explanations may have applicability and relatability to other rural contexts outside NSW and outside Australia.

The aims of this synthesis were:

1. To describe and understand the contexts in which rural health research training occurs and the outcomes of research capacity building endeavors in the rural health workplace.

2. To use these outcomes in context to theorize what mechanisms exist in the education of research for the rural workforce which have led to the kinds of outcomes we see.

3. To develop key principles to guide the development of rural research capacity building programs.

## 2 Materials and methods

This paper brings together five papers centered on rural research capacity building (Table 1) in the form of a realist synthesis. Unlike a traditional systematic review which takes a broad view of the available literature, this realist synthesis uses purposively selected studies to form a unique data set that can be explored using realist principles to extract a new understanding. This approach is underpinned by the concept that the papers present theories about what works for who in what circumstance and that by synthesizing together these theories the underlying causative mechanisms can be unveiled in what is otherwise a complex area (56).

**TABLE 1:** Papers included in synthesis (103).

Referenes	Study type	Location	Number of participants
Schmidt (52)	Single case study	NSW (two organizations)	Single case
Schmidt and Kirby (33)	Cross-sectional study	NSW, Victoria, Northern Territory (multiple organizations)	20
Schmidt et al. (53)	Content analysis	NSW (single organization)	N/A
Schmidt et al. (54)	Qualitative study	NSW (single organization)	18
Schmidt et al. (55)	Qualitative study	NSW (multiple organizations)	22

For this synthesis, the included papers report on the context of research training and the outcomes of the RRCBP and another similar program conducted in a more remote part of NSW and other states. These studies were selected for a number of reasons. Firstly, the RRCBP provides the longest-running example of rural research capacity building in Australia (29, 55) and as such provides opportunity for learnings that cannot be gleaned from shorter-term programs. Secondly, understanding the context in which these programs is an important part of realist synthesis (56) and the authors’ positions as “informed insiders” within these programs allows a nuanced perspective that an outsider may not achieve. Including papers that explore similar programs to the RRCBP or similar contexts to which the RRCBP is conducted provides a diversity of sources, paralleling the fact that rural communities are not homogenous (2). Bringing these studies together creates a necessary richness to the data.

The process of realist synthesis involves synthesizing the findings from the individual studies into new generative mechanisms and was led by the first author (DS). The process commenced with extracting elements known as Context, Mechanism and Outcome statements (CMO) of each of the included studies. These statements (also known as CMO chains) were broken down into their individual elements such as observations on the Context of the rural research capacity building endeavor or the proposed Mechanism that explains observed Outcomes within the study. The elements were considered separately, that is all Context statements were combined to create a collective understanding of Context, and so forth for Mechanisms and Outcomes. These collected components were then reviewed in light of existing literature, particularly through the structural levels of capacity building; individual, team, organizational and supra-organizational (57, 58). These elements were then combined via an iterative and intuitive process of retroduction and hypothesis building to form new CMO chains, using the “creative imagination” described by Bhaskar (59). Critical realism tenets of stratified reality, agency and structure were applied, along with external literature, as mechanisms were hypothesized, discussed between the authors and then explored for logic and coherence, leading to proposed mechanisms being refined, adopted or abandoned. These mechanisms were tested in two ways. Two external experienced health research educators checked the proposed mechanisms for coherence (60) and the consistency of proposed mechanisms with their knowledge and experience of rural research capacity building. Feedback was used to refine the mechanisms and the way they were expressed in CMO chains. Mechanisms were then compared to those highlighted by Cooke et al. (61), in their realist synthesis, a work that applies realist principles to the area of research capacity building but importantly does not consider the rural context. Bracketing and reflexive conversations with the second and third authors (EW and DL) added further rigor to the process (62–64).

Central to this process was the knowledge inherent in being an embedded insider with a deep understanding of the rural health context, the NSW public health system and the process of research capacity building. This insider perspective provided a credible foundation from which creative imagination could be employed. This process was repeated multiple times until a suite of proposed mechanisms were compiled. These CMO chains were then tested and consolidated into practical understandings of what works where and for whom, which could then be transferred into practical recommendations for health system, health service and education purposes.

### 3 Results

Context and outcomes for rural research training within rural NSW were explored at the individual, team and organizational structural levels at which research capacity building occurs (57, 58). While the supra-organizational context is acknowledged, none of the included papers focused on this structural level and it was therefore not a focus in this analysis.

### 3.1 Individual contexts and outcomes

Rural clinicians, with the challenges of rural health service delivery, see not only problems but also opportunities for research investigation (52). Rural individuals experience limited operational planning for research, which can act as an inhibitory structure for research (53). Rural clinicians want rural research to be immediately useful (55).

The individual rural clinician context and the way the individual interacts with that context is constantly evolving. Developing research experience and capability contributes to increased confidence in rural individuals (55), which can translate to changes in their individual agency, or ability to take action within their context. Distributed research training in the workplace can keep experienced health professionals in their roles whilst building research experience (55). It should be noted that not everyone who learns about research wants to continue to apply research skills in their work role (55).

Obtaining organizational support for research training and ongoing research activity where individuals can use their new skills is challenging (55). Without organizational support, a disconnect between workplace and individual can arise where research is seen as an individual pursuit unrelated to organizational goals (54).

The context for individual learning includes a low base of research activity and limited research expertise in rural areas (53). Research training generally needs to be introductory in nature, matched to the learner’s needs (65), and supported by expertise from experienced researchers where available (54). Many clinicians in rural NSW have existing research-relevant skills, so training may build on existing project management or quality improvement skills (52) and may require a multilevel training strategy (58).

Training programs have shown increased individual research experience (28, 33) and a range of research-specific and transferable skills such as project management experience, enhanced critical thinking, improved communication and improved confidence (55).

Close-to-practice research, such as that completed in experiential rural research programs (33, 55), is a key enabler of capacity building (29, 57, 58). This research activity, along with changes in individual research capability, skill and experience amount to real-world research capacity building (66) and continue to demonstrate that individual training can have capacity building outcomes (29).

### 3.2 Team contexts and outcomes

The papers synthesized focused on the organizational (53, 54) or individual level (33, 52, 55) and provide fewer insights into the team context. A supportive work team is an important facilitator of research (67), and the attitudes of work colleagues can be a powerful structure that influences rural clinicians undertaking workplace-based research. Research-emergent and novice clinician-researchers can make their own research networks to provide team level support that their workplace team may not (55).

Building a more capable, confident and skilled worker as a result of research experience and research training has team benefits including improved evaluation rigor, raised profile of research within a team, creating research activity and retaining a skilled workforce (55). Building team research capacity may be cumulative, with the collective individual capability outcomes contributing to team level capacity.

### 3.3 The organizational level

Research conducted within rural health organizations in NSW is often not conducted for or by these organizations (53), perpetuating the perception that research is not something that rural health organizations can do: a “too rural and too poor” view (54). The perception of limited capacity is both an outcome of the limited health research expertise within rural health organizations (12) and the difficulty accessing research funding (68), and is also a mechanism of limited research activity. This co-occurring role of both cause and outcome reflect a stratified reality: organizations that see themselves as incapable of undertaking their own research may engage with outside organizations in a passive way, thus limiting opportunities for research capacity building within the organization.

External partnerships are a potential solution to limited access to research expertise in rural areas (12, 54). These collaborative approaches across sectors, either formally or informally, can help provide access to research knowledge and support that is vital for research capacity development.

The organizational context for those wanting to learn about research includes the ability to access practical support, such as operational planning or positional responsibility for research (53), valuing and promoting research endeavors (54) and organizational commitment (55). Practical support may include creative solutions such as incorporating research activity into routine work to offset a lack of funding (52).

The value placed on research in rural health organizations may vary between strategic and operational levels (53) and a mismatch between organizational language and actions concerning the value of research activities (55) can create an inhibitory influence on rural health staff. A perception that research is a low-value individual activity inhibits the uptake of research opportunities and learning in research (54). Demonstrating that research is valued is a key facet of research capacity building (57, 58, 61).

An organizational perception that research is an individual, rather than an organizational, activity can lead to research activity being driven largely by the agency of individuals (53, 54). This reliance on individual agency is associated with a limited number of nursing and allied health research projects (53).

Maintaining research activity outside of the supportive structure of training programs also relies upon the individual agency of the worker (55), although there is a limit to how much individual agency can overcome structural limitations. A mismatch between research capability and research capacity can lead to discontent (55).

Organizational outcomes resulting from research training include increased local research activity, dissemination of research findings, demonstrable leadership and the establishment of partnerships (29, 33, 49, 54, 55).

Research training can be viewed as an organizational investment rather than a cost burden (52), if the organization acknowledges that retaining experienced staff whilst improving policy and practice (55) is a real return on that investment. Research-trained clinicians demonstrate capability as an outcome of a capacity building endeavor, whilst being an enabling mechanism of research activity by assuming the roles of researcher, resource person or mentor.

### 3.4 Synthesis and new generative mechanisms

This new understanding of outcomes in context reveals generative mechanisms that underlie research capacity building for the rural health workforce. A suite of proposed mechanisms were derived and are expressed as CMO chains in Table 2.

**TABLE 2:** Generative mechanisms of research capacity building in rural health workplaces expressed as context, mechanism outcome (CMO) chains (103).

Context	Mechanism	Outcome
In rural health services	A perception that the organizations are “too rural and too poor”^†^ to undertake research	Limited research activity
Where individual and organizational rural research capability exists	Limited operational planning for, organizational prioritization of, and valuing of research	Limited engagement with research and a reliance on individual agency as a primary driver for internal research activity
Viewing research as an individual activity	A disconnect between individual and organizational research priorities	Research seen as being of low organizational value
Rural health workers with an interest in research are seeking to build on their existing skills, and seek an introductory level of education and support by experts in research	Limited access to research expertise in rural health services	A need for collaborative approaches where health services and training organizations, research units and universities create mutually beneficial relationships
Where there is little funding to create formal partnership arrangements	Informal collaborations based on existing relationships	Collaborations that are contingent upon goodwill, flexibility and mutual goals
When research capacity building activities occur in rural health services	The experiential nature of research capacity building programs used in rural Australia	Builds individual skill, increases research activity, and produces research that changes practice
Where research capacity building programs are designed to upskill individuals	Training delivered at an individual level	Produces changes primarily at the individual level and primarily in capability, with fewer team and organizational benefits
When undertaking research capacity building with rurally-based health workers	Structural solutions such as the design of program, creative ways to enable protected research time, and strategic engagement with the hosting organization	Can overcome some of the inherent limitations which include a small, dispersed workforce, lack of organizational support and limited funding
Where introductory research training has been undertaken	Limited opportunities and structural inhibitors	Reinforce that individual skill development is important, but not sufficient for ongoing independent research

^†^Schmidt et al. (54).

Perceptions and beliefs can exert influence (69), and in a critical realist sense are therefore real. In rural health services a real underlying perception that the organizations are “too rural and too poor” to undertake research can lead to empirical limited research activity (54). Beliefs can, at an organizational level, stifle new approaches (70) and exert a real influence on rural health research. Beliefs and knowledge can intersect as explanations of the social world (71), and while there is evidence rural organizations are empirically disadvantaged in research grant funding (68), it is the real perception that rural health organizations are inherently incapable of undertaking research that is a key driver of low levels of research activity.

While research capability is an important part of research capacity within rural health services, limited operational planning for research, low organizational prioritization of research, and a perceived low organizational valuing of research directly are factors that impact on engagement with research. In rural NSW, Australia, a clear gap in organizational planning for research (53) has led to a reliance on individuals, and individual agency, to drive research activity. This resulted in research being seen as an individual activity disconnected from organizational research priorities and thus of low organizational value. The alignment between individual and organizational priorities for research is critical, given that a lack of organizational support leads to limited research activity (55) and is a cause of withdrawal from research training programs (34).

Limited research expertise in rural health organizations (54) is a mechanism leading to a need for collaborative approaches to provide both introductory research education and expert support for research. Collaborative approaches are essential for providing access to expertise, particularly when the rural context is considered (17, 23, 31).

Rural partnership arrangements are often relationship-based (33) and are contingent upon goodwill, flexibility and mutual goals. Mutual goal-setting is an important part of collaborative approaches to research partnerships (72) and in rural areas these relationships can be effective where the goals of the workplace and the learning institution align (54).

When training in research occurs in rural areas the experiential nature of training programs used in rural NSW builds individual skill, increases research activity, and produces research that changes practice (33, 55). Experiential learning aligns closely with adult learning principles (73, 74).

Training programs delivered at an individual level lead primarily to individual outcomes, with fewer team and organizational benefits. It must be noted that the tools used to empirically assess outcomes, such as the research spider (75) which is commonly used in assessing research experience (28, 76), are aimed at the individual level so could fail to identify co-occurring team and organizational outcomes. Team research capability and culture are less well developed in rural areas than individual or organizational capability (77) and thus an avenue for future research capacity building in rural areas should focus on team approaches as have been trialed elsewhere (19, 78–81).

Some of the inherent limitations of rural research education, such as a small, dispersed workforce, lack of organizational support and limited funding, can be accommodated by structural solutions such as the design of distributed training programs (33), creative ways to enable protected research time (17, 52) and strategic engagement with the hosting organization (17, 33).

While receiving training in research equips health workers for ongoing research activity (28, 29, 55) limited opportunities to use these skills and structural inhibitors such as a lack of time and resources reinforce that individual skill development is important, but not sufficient for ongoing independent research. This demonstrates the difference between research capability and research capacity; trained clinician-researchers *could* undertake independent research, but this does not mean that they *can* undertake independent research.

### 3.5 Testing research capacity building theory

The proposed mechanisms, after testing for coherence (60), were compared to the mechanisms of research capacity development proposed by Cooke et al. (61) in their realist synthesis (see Table 3). The mechanisms described by Cooke et al. represent a “best evidence available” model, one that is unencumbered by the constraints of the rural environment. This allows a comparison of “what is” in the rural environment of NSW, to “what could be” in Cooke et al.’s model. Demonstrating concordance between the two models confirms that these proposed mechanisms derived in this synthesis display coherence, whilst allowing an exploration of the rural and non-rural differences of the two models.

**TABLE 3:** Emerging mechanisms from this synthesis contrasted with mechanisms of research capacity development proposed by Cooke et al. (61, 103).

Research capacity building mechanisms		Mechanisms of research capacity development^†^
A perception that the organizations are “too rural and too poor” to undertake research****A limited amount of research expertise in rural areas	*Contrasts with*	Modeling positive behaviors
Limited operational planning for, organizational prioritization of, and valuing of research	*Contrasts with*	Signaling importance
A disconnect between individual and organizational research priorities****Limited opportunities and structural inhibitors	*Contrasts with*	Exceeding the sum of the parts
Informal collaborations based on existing relationships	*Aligns with*	Exceeding the sum of the parts Coproducing knowledge
The experiential nature of training programs used in rural Australia that build individual capability, increases research activity, and produces research that changes practice	*Aligns with*	Learning by doing Feeling that you are making a difference
Structural solutions such as the design of program, creative ways to enable protected research time, and strategic engagement with the hosting organzsation	*Aligns with*	Releasing resources Liberating the talents

^†^Cooke et al. (61).

Notably, the mechanisms proposed in this synthesis contrast with those of Cooke et al. (61) in role modeling, signaling importance (where individuals see that engaging in research is a valued part of the organization’s business) and exceeding the sum of the parts. In the rural context, a shortage of rural researchers and organizational commitment lead to limited role modeling and limited visibility of research (53, 54). This contrast highlights that rural and metropolitan approaches to research capacity building differ due to important structural influences, such as geographical spread of the workforce, availability of research experts and ability to access to research funding. While the increasing the number of rural researchers is a long-term solution, rural health organizations can influence other elements such as increasing the visibility of research and signaling its importance within rural health organizations.

The final aim of this synthesis was to use these mechanisms to develop general principles to guide the development of rural research capacity building programs. These are found in Table 4. These principles extend the mechanisms into useful actions that can be applied in rural health contexts.

**TABLE 4:** Key elements and strategies to optimize rural research capacity building (103).

Key element	Strategy
For individuals	Systems should be implemented to identify research-interested individuals whose research interests match the health organization’s research plan. Offering short training courses on research related topics like systematic reviews or evidence-based practice can be a way of identifying these individuals.
	Assessment of learning needs, the characteristics of the team in which they operate and levels of organizational support should be undertaken. This ensures training matches the learning needs of the individual, delivers education appropriate to the learner’s context and ensures the organization is capable of, and willing to, create an environment conducive to the development of research capacity and activity.
For teams	Rural research training should integrate skills for teamwork and relationship building into learning experiences. This will emphasize the importance of researching in teams and the value of collaborative relationships.
	Specific research training for teams that is developed, trialed, and evaluated is a strategy that can be implemented.
For organizations	Rural health organizations should have a clearly defined and communicated research plan that explicitly includes investment in research capacity building and that will allow researchers and training organizations to align their activities and goals for mutual benefit.
	Dedicated roles and resources via a research office that ensures ongoing coordination and commitment by senior leaders within the health organization to ensure capacity building is progressing in line with organizational planning.
	Formal partnership arrangements between the organization in which the trainee works and any external partnering body should be implemented to develop structures to support the development of research capacity.
For educators	Training in research methods should be underpinned by capacity building principles, as the short and long-term outcomes for programs built on this platform are evidenced within the literature. If another specific educational theory is applied, this should be explicitly named to allow future study of the outcomes from alternative educational theories.
	Peer support, mentoring and supervision aspects are critical. The risk of social isolation for rural researchers can be mitigated by developing opportunities for connection between research capable people within and beyond any research capacity building program. These connection processes can be extended to link those with a research interest to those with research capability.
	Given the importance of informal relationships, continuity and consistency in training delivery is needed. The design of research education can provide continuity by avoiding short-term funded projects and favoring long-term partnership-based approaches.

## 4 Discussion

Embedding academic researchers into rural health services provides a number of solutions to problems within rural organizations (82). Given the challenges associated with attracting and retaining rural academics (83), the idea of creating research-capable rural health workers that function as clinician-researchers is appealing. The “train them where you need them” philosophy has been shown to be instrumental for building and retaining rural workforce in health services such as medicine, nursing and allied health (84–87). Applying this principle to research, developing research experience and research capacity within rural health services will lead to rural-relevant research that leads to improved healthcare for rural communities.

There is no single model of research capacity building that can be applied across rural environments. While distributed programs built on a capacity building framework (28, 29, 55) are seen within the NSW context, programs built on other foundations also aim to build elements of research capability such as experience and research skill, or increase both research capability and research capacity in individuals (65, 76, 88–90). Educational philosophy is a component of research capacity building literature which did not emerge as a causative mechanism within this synthesis. Ensuring that educational foundations are described in future studies would allow exploration of alternate foundations and the outcomes of these educational approaches.

Educational and capacity building approaches are most effective when they incorporate experiential learning (61). Experiential elements extend the learning experience from building capability to building capacity, simply by the act of doing. From a learning point of view, “doing” or applying knowledge demonstrates a greater level of expertise than “learning about” (91).

Those building health research capacity must account for the unique characteristics and context of the rural environment; the organization’s goals, the individual’s position within the organization and the willingness of the organization to tangibly support the individual through funding or protected time for research. Models may need to be designed with a structure that assists learners in overcoming rural or remote challenges, including programs designed to reduce isolation for learners (33), as reduced isolation is associated with completion of researcher training in rural areas (34).

As there is limited research expertise within rural health services, capacity building approaches must include partnering for expertise. Partnering may be internal within the organization or with an external partner such as a university (92), but must be mutually beneficial and without the rural health organization ceding control of the direction of the research (54). Maximizing the value in existing relationships using a collaborative approach is a logical means of extending support. Rural universities have a role in researcher development through higher degree programs, and have undertaken a range of collaboration approaches with health services (92). The articulation between university-based and workplace-based training approaches is an area for further exploration.

Alongside this experiential component there is a need for targeted education. Given the limited critical mass of research expertise in rural areas this education is likely to be introductory research methods. However, “liberating the talents” (61) may mean that research education builds on existing skills rather than assuming all rural staff are commencing as novices. An assessment of learning needs should enable educational opportunities at the required level, rather than a generic approach.

Being an informed consumer of research that knows how to understand and apply research as part of evidence-based practice does not mean that all rural clinicians need to be capable of undertaking a research project. Selecting individuals for research capacity building opportunities should balance the passion of the individual and the needs of their organization. A committed and enthusiastic individual may become a valuable independent researcher given the right support.

Delivering training at the individual level will produce primarily individual level outcomes. Despite limited evidence of team approaches to research capacity building, a team approach does present as a structural solution to the risk of isolation for rural health workers undertaking research. Training in teams also maximizes the use of existing expertise with a rural team, again “liberating the talents” (61).

In addition to formal learning, research capacity can be enhanced through peer learning, with those who have research experience taking a role in building research activity and capacity with those around them (55). Learning about research by interacting with others who are undertaking research can be a form of cultural constructivism (33, 93), where a sense of belonging in the world of research is constructed through interaction and immersion, as well as activity and education. Connecting research capable and research interested individuals is a means of providing a supportive environment.

Extending capability into research capacity is more than an educational endeavor (94). As a health system the focus has often been on developing the motivated individual without the accompanying supportive environment (94). Structural supports (54) and meaningful opportunity to conduct research post-training (55) are essential to addresses the limited opportunities and structural inhibitors that prevent the transformation of research capability into research capacity. An organization committing to developing a rural health worker into a clinician-researcher should make a similar organizational commitment to creating conditions in which a clinician-researcher can function as both clinician and researcher. This combination of “smart and motivated people positioned in supportive environments that allow(ed) them to ask hard questions and pursue hard problems” is the key to success for clinician-researchers (95).

Delivery of research capacity building programs or approaches by those internal to the health system may influence the development of these supportive environments in a way that external education providers cannot. While informal relationships are a cornerstone of rural collaboration, training providers from outside the health system may need to partner with rural health organizations in formal agreements in addition to reciprocity and mutual respect (54). Continuity is important to building relationships, and consistent long-term offering of a research capacity building program (55) adds to this continuity.

Extending this concept of organizational commitment, there needs to be an operational responsibility for research (53). Aligning individual research effort to organizational direction is needed (54). The provision of a clear research direction for the rural health organization, one that is signaled as important (61), will allow clinicians to align their own research agenda to that of their organization. Specific research directions within an organization can allow external education partners to align teaching goals and processes to this direction. This allows effective partnering without the health organization ceding control of the direction of research activity and education (54).

Urban-centered research conducted in rural areas does little to enhance research capacity in the rural workforce, and ensuring urban-developed research has a rural individual as part of the project team is a practical capacity building step. This brings rural staff into contact with experienced researchers, thus maximizing the rural benefit of this research activity (53). Other models such as embedded researchers may also provide a more engaged organization (22, 26, 82). Similarly, a more egalitarian funding model that emphasizes partnerships may provide greater capacity building potential (18).

Lastly, organizations can address the “too rural and too poor” perception by adjusting the way in which they perceive and value research. Structures such as the hierarchy of evidence (96) and the way in which small-scale clinician-led research has been gradually subsumed by larger research driven by networks (97, 98) may lead rural organizations to view smaller, clinician-led projects to be of little or no value. Supporting, acknowledging, valuing and celebrating these smaller projects, “signaling importance” (61), can add to real-world research capacity. This small-scale research capacity can become a foundational building block for larger future research activity or for collaboration with a larger research organization.

In NSW the combination of individual LHDs with a centralized “system manager” (51) presents an opportunity for an educational body within the central system to interact with and influence the individual rural organizations. Australian states with a greater or lesser degree of centralization will have different challenges. Internationally the difference in health funding models and health system structures should also be acknowledged.

This synthesis has considered factors at three of the four structural levels of capacity building; individual, team and organizational (57, 58). Additional research considering the supra-organizational level, that is system-wide and policy factors, again from a critical realist perspective, would add another layer of depth in addition to the levels explored in this synthesis. This would be possible only in a program or programs that have been running for a sufficient length of time for outcomes of this type to be realistically achieved. Other future research directions include expanding team-level rural research capacity building approaches started in Queensland (19) and a nuanced economic evaluation of a capacity building program for rural areas which explores the longer-term value of investment in research capacity building from a business perspective. Research training, along with valuing and supporting research at multiple levels of an organization can lead toward a research-supportive culture (53). Research culture is often measured by self-report (45, 77, 99). An ethnographic study exploring the impact on research culture within a team as a result of training individuals in research would provide valuable insights and could be incorporated into a critical realist perspective (100).

### 4.1 Strengths and limitations of this synthesis

This synthesis draws from five papers containing small sample sizes, which may be considered a limitation. The size of these studies are consistent with similar studies in this field, and the studies themselves have samples representative of their trainees drawn from organizations typical of large rural health services within NSW. The focus on the NSW, and similar, contexts may limit generalizability however the nature of realist approaches is to explore what works for who in what circumstance (101). A broader approach which includes a wide range of contexts would in turn limit the ability to derive new understandings using realist approaches.

Drawing data from a single body of work may be seen as a potential limitation. The primary author’s position is as an insider who works in rural health research. This can be both a strength and limitation, with this insider perspective and inherent knowledge allowed for nuanced exploration and the expertise to apply retroductive processes and develop explanatory mechanisms of “why things are as they are” using critical realism (102). Offsetting these strengths is the limitation inherent with the personal biases brought by the researcher. Reflexive practices and consistent application of bracketing were used to enhance the rigor of this synthesis.

The diversity of source and approach enabled by different organizations, research types and data collection methods is a strength of this synthesis. Combining these as a single body of work in this synthesis is made possible by the use of critical realism, which embraces diversity in research methods.

## 5 Conclusion

This synthesis of studies focused on rural research capacity building has revealed a range of mechanisms including prevailing attitudes toward research, limited organizational valuing of research and a disconnect between individual and organizational research priorities, along with limited access to research expertise in rural health services. These inhibitory mechanisms are countered using existing relationships to build informal collaborations within teams and organizations to maximize the use of current expertise.

Distributed research education is important but not sufficient alone to develop rural clinicians into clinician-researchers. Structural supports are needed within rural health organizations, including organizational commitment to create environments in which rural health workers can learn about research, develop research experience and opportunities to undertake research. It is this combination of research training and supportive environments that will lead to optimized rural research capacity.

Capacity building endeavors should carefully consider the learner context and commit to long-term relationship-based approaches to rural research training programs. Further structural solutions such as the design of distributed education programs, creative ways to enable protected research time, and strategic engagement with the hosting organization are important aspects of research capacity building.

Individual level interventions have driven individual level outcomes. These are important but may miss opportunities to maximize the potential to move from individual capability to individual and organizational research capacity in rural health organizations. Considering and addressing structural supports will not only inform the next stage of organizational investment but will maximize the benefits of distributed training for rural research capacity building.

## Data Availability

The original contributions presented in the study are included in the article/supplementary material, further inquiries can be directed to the corresponding author.
